# Supplementary material for: Impact of Different Diagnostic Procedures on Diagnosis, Therapy, and Outcome in Horses with Headshaking: Recommendations for Fast-Track Advanced Diagnostic and Therapeutic Protocols
Source: Animals (Basel). 2022 Nov 13;12(22):3125. doi: 10.3390/ani12223125 (PMC9686903; doi:10.3390/ani12223125)
Supplement: Supplementary file 1 [file animals-12-03125-s001.zip › animals-1971575-supplementary.pdf]

Table S1: Diagnostic Score. Every conducted diagnostic procedure was scored depending on the highest severity of findings for one individual horse. All the scores for one horse were added and divided through the maximum reachable points, leading to a total diagnostic score for every horse [%].

**Findings in diagnostic procedure**	**Score**
Without finding	0
Mild finding	1
Moderate finding	2
Severe finding	3
**Total diagnostic score per horse [%]**	$\frac{\Sigma \text{ scores from every conducted procedure}}{\text{maximal reachable points}}$

Table S2: Prognosis Score. Outcome of clinical signs, outcome of equestrian use, and responsiveness to therapy are scored for every individual horse. All the scores for one horse were added and divided through the maximum reachable points, leading to a total prognosis score for every horse [%]. The prognosis score was only calculated for horses with sufficient information about the outcome regarding clinical signs, equestrian use, and responsiveness to therapy.

**Outcome of clinical signs**	**Score**
No clinical signs	2
Improvement	1
Unchanged	0
Deterioration	-1
Euthanasia due to headshaking	-2
**Outcome of equestrian use**	**Score**
Improvement	1
Unchanged	0
Deterioration	-1
Euthanasia due to headshaking	-2
**Responsiveness to therapy**	**Score**
Improvement	1
unchanged	-1
**Total prognosis score per horse [%]**	$\frac{\Sigma \text{ scores for course of symptoms, equestrian use and response to therapy}}{\text{maximal reachable points}}$

Table S3: Overview of findings within the diagnostic investigation. Absolute abundance of findings is shown for conducted diagnostic procedures. Given the fact that a horse can have more than one finding within a single diagnostic procedure, abundance of findings can exceed the total number of horses.

Diagnostic procedure	Abundance of findings (n)
General clinical examination (mainly heart murmurs, thickened mandibular lymph nodes and increased vesicular breathing sounds)	
• Mild finding	41
• Moderate findings	8
Clinical neurological examination	
• Gait abnormalities	22
• Hyperaesthesia of the face	16
• Findings within the cranial nerve testing	10
• Further findings	25
Ophthalmological examination	
• Chorioretinopathy	20
• Cataract	17
• Epiphora	12
• Findings within the vitreous body	12
• Further findings	28
Upper airway endoscopy	
• Increased mucus in the nasal passages	148
• Hemiplegia laryngis sinistra	91
• Pharyngeal lymphoid hyperplasia	67
• Thickened temporohyoid articulation	60
Lower airway endoscopy	
• Suspected Equine Asthma	44
Oral examination	
• Hooks, edges, ulcers	158
• Infundibular dental caries	65
• Diastema	32
• Tooth fracture	28
• Gingivitis	22
• Opened pulphorns	18
Radiographs of the head (anatomic region of findings)	
• Cranial attachment of the nuchal ligament	73
• Teeth	24
• Paranasal sinuses	8
• Infraorbital channel	3
• Temporohyoid joints	3
• Further findings	1
Computed tomography of the head (anatomic region of findings)	
• Cranial attachment of the nuchal ligament	65
• Infraorbital channel	54
• Teeth	33
• Temporomandibular joints	27
• Paranasal sinuses	25
• Temporohyoid joints	22
• Ears	10
• Further findings	10
Magnetic resonance imaging of the head	
• Otitis media	3
• Arthropathy temporomandibular joint	3
• Sinusitis frontalis	2
• Cholesterolgranulomes	2
• Temporohyoidosteoarthrosis	1

• Otitis externa<br>• Further findings	1<br>4
Orthopaedic examination<br>• Lameness grade 1/5<br>• Lameness grade 2/5<br>• Lameness grade 3/5<br>• Further findings	 19<br>17<br>1<br>3
Clinical examination of the thoracolumbar spine<br>• Mild pain or decreased flexibility<br>• Moderate pain or decreased flexibility<br>• Severe pain or decreased flexibility	 34<br>13<br>2
Radiographs of the thoracolumbar spine<br>• Kissing spines<br>• Osteoarthrosis of the intervertebral joints<br>• Further findings	 22<br>1<br>4
Radiographs of the cervical spine<br>• Arthrosis of cervical articular process joints<br>• Osteochondral fragments of cervical facet joints<br>• Further findings	 23<br>9<br>15
Computed tomography of the cervical spine<br>• Arthrosis of cervical articular process joints<br>• Osteochondral fragments of cervical articular process joints<br>• Further findings	 9<br>4<br>5
Otoscopy<br>• Increased amount of cerumen and debris<br>• Otitis externa<br>• Proliferations within the external ear channel	 20<br>3<br>2

Table S4: Results of laboratory analysis. Relative abundance of horses within reference range is shown [%]. From those horses that are outside the reference range, the average value plus standard deviation is presented.

Parameter	Refer-ence range	Horses within reference range: relative abundance [%]	Average value of horses outside reference range	Standard deviation	Absolute number of horses measured (n)
Haematocrit [%]	30-45	93	**45,31**	4,40	192
Erythrocytes [T/l]	5-10	99	**10,72**	0,58	188
Haemoglobin [g/l]	110-170	99	145,75	40,51	188
Thrombocytes [G/l]	90-300	94	**66,36**	26,90	172
Fibrinogen [G/l]	1,8-5	73	3,47	3,06	86
Leukocytes [G/l]<br>- Segmented neutrophils [%]<br>- Lymphocytes [%]<br>- Monocytes [%]<br>- Eosinophils [%]<br>- Basophils [%]	4,3-12<br>45-70<br>20-45<br>0-5<br>0-4<br>0-2	92<br>76<br>85<br>86<br>93<br>99	5,71<br>**70,66**<br>27,77<br>**6,39**<br>**5,11**<br>**8,40**	2,62<br>14,56<br>17,91<br>3,32<br>1,75<br>4,88	192<br>150<br>150<br>129<br>137<br>127
Glutamat dehydrogenase [U/l]	0-6	81	**80,37**	131,28	131
Alkaline phosphatase [U/l]	0-290	100			132
Aspartate aminotransferase [U/l]	0-170	68	**249,39**	81,29	156
Gamma glutamyl transferase[U/l]	0-20	72	**53,38**	38,91	156
Creatine kinase [U/l]	0-130	77	**181,97**	68,76	157
Lactate dehydrogenase [U/l]	0-235	88	**321,37**	70,43	155
Sodium [mmol/l]	125-150	99	135,90	14,90	152
Potassium [mmol/l]	2,33-4,18	90	3,22	1,10	151
Calcium [mmol/l]	1,45-1,6	83	1,58	0,42	151
Chloride [mmol/l]	95-105	80	**107,77**	6,59	151
Magnesium [mmol/l]	0,5-0,9	93	**1,00**	0,21	122
Bicarbonate [mmol/l]	20-28	4	27,99	1,88	27
Base excess	-3 - +3	64	**4,20**	0,64	28
pH	7,38-7,42	53	7,38	0,04	105
Phosphorus [mmol/l]	0,7-1,5	89	0,58	0,00	9
Bilirubin [µmol/l]	8,9-49,9	96	44,27	16,32	140
Bile acids [µmol/l]	0-12	94	**17,96**	7,83	84
Blood urea nitrogen [mmol/l]	0-6,8	98	**7,08**	0,20	139
Creatinin [µmol/l]	0-160	99	**189,50**	6,50	139
Triglycerides [mmol/l]	0-0,6	100			141
Albumin [g/l]	27-40	93	34,09	2,89	109
Total protein	54-78	97	77,26	1,94	186
Glucose [mmol/l]	4-8	75	6,58	0,94	160
Lactate [mmol/l]	0-0,7	46	**0,95**	0,25	158

Table S5: Summary of conducted statistical data analysis

Diagnostic score

Number of horses: 122

Anova Table (Type II tests)

Effect	X^2	Df	F value	p-value	
Prognosis score	0.09664	8	0.979	0.3751	ns

Prognosis score

Kruskal-Wallis rank sum test

Effect	n	X^2	Df	p-value	
Diagnosis	92	8.8627	8	0.354	ns
General clinical examination	122	3.1338	8	0.9257	ns
Ophthalmological examination	103	4.9943	8	0.7582	ns
Clinical neurological examination	98	5.2997	8	0.7251	ns
Orthopaedic examination	43	16.625	7	0.01998	*
Clinical examination of the thoracolumbar spine	47	2.2382	7	0.9455	ns
Oral examination	109	7.4494	8	0.489	ns
Upper airway endoscopy	102	8.7985	8	0.3596	ns
Lower airway endoscopy	104	10.72	7	0.1513	ns
Radiographs head	110	15.626	8	0.04805	*
Radiographs cervical spine	94	3.3874	7	0.847	ns
Radiographs thoracolumbar spine	31	9.648	7	0.2094	ns
Otoscopy	82	6.4995	8	0.5915	ns
CT head	95	9.9838	8	0.2662	ns
CT cervical spine	27	3.1628	6	0.7882	ns
MRI brain and adjacent structures	70	7.5957	8	0.4739	ns

Pairwise comparisons for 'orthopaedic examination':

Wilcoxon rank sum test with continuity correction

Comparison		p-Value (FDR corrected)	
No finding	Mild finding	0.140	ns
No finding	Moderate finding	0.048	*
Mild finding	Moderate finding	0.343	ns

Pairwise comparisons for 'radiographs head':

Wilcoxon rank sum test with continuity correction

- ➔ Not significant (p = 0.81)

Responsiveness to therapy (Figure 3)

Number of therapies: 159

Pearson´s Chi-squared test with simulated p-value

X^2 = 12.52, df = NA, p-value = 0.05347
